# Factors Enhancing Trust in Electronic Communication Among Patients from an Internal Medicine Clinic: Qualitative Results of the RECEPT Study

**DOI:** 10.1007/s11606-021-07345-9

**Published:** 2022-01-19

**Authors:** Annie Moore, Catia Chavez, Michael P. Fisher

**Affiliations:** 1 Internal Medicine, University of Colorado Anschutz Medical Campus, Aurora, CO USA; 2 University of Colorado School of Medicine, Aurora, USA; 3 ACCORDS, University of Colorado Anschutz Medical Campus, Aurora, CO USA; 4 Department of Health Sciences, Towson University, Towson, MD USA

**Keywords:** Patient trust, Patient engagement, Patient portal, Telehealth, Electronic health record, Patient secure messages

## Abstract

**Background:**

Electronic health records are now the norm in US healthcare. Bidirectional patient portals allow frequent communication between patients and their healthcare team. Many studies have examined the importance of patient engagement and trust between patients and their healthcare team, typically in the context of face-to-face interactions. Little is known about how patient trust and engagement are built or enhanced through electronic communications. COVID-19 provided a unique time in history for this novel exploration.

**Objective:**

Our objective was to learn how patients experience trust formation through electronic communication (patient messaging and video visits) with their healthcare team.

**Design:**

Our research was guided by grounded theory methodology. Qualitative interviews were conducted between February and December 2020 with patients or their caregivers from an internal medicine clinic in Colorado.

**Participants:**

Fifty-one participants were recruited by age group and gender to represent the clinic’s adult ambulatory care demographics. Seven were patients’ caregivers who were purposefully recruited. Average age was 53 with an educated, middle class, and largely white predominance in our eventual sample.

**Approach:**

Thirty-minute semi-structured interviews were conducted using an interview guide informed by a validated physician-patient trust scale. Interviews were conducted by telephone, recorded via Zoom, and transcribed. Results were analyzed and coded in ATLAS.ti utilizing the constant comparative method, with two coders.

**Key Results:**

Patients experienced enhanced trust in their healthcare team through electronic communications. Interpersonal and system factors contributed to trust formation. Promptness of reply was the most salient factor in trust formation with a majority desiring same day response.

**Conclusions:**

Patients now rely on electronic communication with their healthcare team. Opportunities exist to leverage this to improve health outcomes. Important research in expanded demographic groups, along with ambulatory healthcare redesign, will be necessary to optimize benefits of electronic communication with patients and meet patient expectations.

**Supplementary Information:**

The online version contains supplementary material available at 10.1007/s11606-021-07345-9.

## INTRODUCTION

Electronic health record (EHR) adoption is now the norm in the USA. Over 90% of US physicians use an EHR in their outpatient clinical environments^1^. Many studies have examined the importance of patient engagement and trust between patients and their healthcare team^2^. These studies are historically based on face-to-face interactions. Little is known about trust formation within EHR portal communication.

Patient portals, defined as an application to “allow patients to interact with their health information and communicate with providers outside the traditional office visit”^3^, are an effective way to improve patient-provider communication as well as patient outcomes^4, 5^. A study by Lyles et al. is the first known US research on the potential power of patient portals to improve trust in care, specifically diabetes care^1^. Sieck et al. identified trust as one of three important psychological benefits of patient portal use for patients with chronic illness^6^.

Although patient portal technology provides opportunities for physicians to engage patients in their healthcare and potentially improve medical outcomes^7^, challenges remain regarding adoption and use of portals. While some patients are keen to use portals to communicate with their healthcare team and access their EHRs, others are more reluctant^5^. Characteristics such as patient socio-demographics and medical condition can be predictors of portal use^3^. The COVID-19 pandemic has impacted patient adoption of video visits and EHR messaging^8–10^.

In this research, part of the broader Role of Electronic Communication to Enhance Patient Trust (RECEPT) study, we explore trust formation and enhancement among patient portal users, during the first year of the COVID-19 pandemic. Our research questions were to (1) determine how portal use impacts trust among patients, either positively or negatively; (2) assess how various portal features influence trust; and (3) explore how trust is experienced differently in three demographic groups.

## METHODS

Conducted February through December 2020, this study involved qualitative interviews with patients or their caregivers, from an internal medicine clinic in Colorado. Our research was guided by grounded theory methodology^11, 12^, a systematic approach capable of providing an in-depth understanding of the complexities associated with patient trust. The study was deemed exempt from human subject protection oversight by the Colorado Multiple Institutional Review Board in August 2019.

### Sampling and Recruitment

Prospective interviewees were recruited via three complementary methods. First, patients were identified through an EHR search based on defined inclusion and exclusion criteria. A physician informaticist provided a technique to sort patients who met these criteria and provided this list to our senior research assistant, who then purposively contacted patients stratified by age group, gender, and physician to provide a broad sample (~1000 patients). A standardized recruitment script was used. Inclusion criteria were (1) patient or caregiver of patient at the internal medicine clinic, (2) portal user defined as participating in portal communication once within the past 12 months, and (3) 18 years or older. Exclusion criteria were (1) employees of the clinic who were also patients and (2) patients of the principal investigator. Second, as caregivers were unable to be identified through an EHR search, physician input was elicited to ascertain these individuals. All identified caregivers were then contacted by phone and/or email using our recruitment script. Caregiver was defined as a family member or entrusted person who accessed the EHR portal as proxy for a clinic patient. Finally, we utilized theoretical sampling to recruit certain participants based on emergent findings^13^. For example, in April 2020, as telehealth experiences became a salient discussion point, we added experiencing a telehealth visit as an inclusion criterion. Thus, interviewees recruited prior to April 2020 might not have had a video visit, while those recruited subsequently were required to have one. This change in recruitment corresponds with organizational shifts during the pandemic, as physicians were transitioned to offer telehealth visits in late March 2020. An incentive of $50 was offered as a gift card. Verbal consent was obtained prior to interviews.

### Data Collection

Interviews were semi-structured, conducted via telephone in English, and approximately 30 min in length. Discussion topics included demographic data; information on past portal use; portal experiences; and patient trust including fidelity, honesty, and protection of privacy. The interview guide was developed by the research team and informed by physician-patient trust scales. We explored five well-known validated scales to understand the known components of patient trust^14–18^. The scale developed by Hall and colleagues^14^, commonly referred to as the Wake Forest Physician Trust Scale, is the primary scale that informed the development and assessment of our interview guide (see appendix). Interviews were audio recorded in Zoom and professionally transcribed. Interviewing ceased when we found the properties of our themes were well established and that additional interviews offered no new information pertaining to these themes or to our research questions. This “saturation point” was met after contacting approximately half the patients who met the pre-specified inclusion and exclusion criteria.

### Data Analysis

Interview data were analyzed in ATLAS.ti version 9.0.20 (Scientific Software Development, GmbH, Berlin) using the constant comparative method^11^. Codes were developed inductively and applied by two team members. The first seven interviews were coded independently by each of the two team members, who met to resolve coding discrepancies through discussion and consensus. The result was a list of approximately 40 initial codes, many with various subcodes, pertaining to patient portal use and trust (see appendix). For remaining interviews, one team member coded all transcripts, while another researcher simultaneously coded one-third of those transcripts to ensure consistency of coding. Concordant processes of coding and memos on codes enabled the elaboration of codes and clustering of codes into categories. Data were then reviewed a second time by a researcher who applied a set of seven focused codes. These conceptually oriented codes had been identified and agreed upon by the research team through the analysis of concepts and patterns within and across the initial coded data. The themes presented in this manuscript correspond directly to the focused codes.

## RESULTS

Overall, 404 patients were approached, and 51 chose to participate. Participants had a mean age of 53 years, were predominantly White (84.3%), and possessed high levels of education (82.4% with a bachelor’s degree or higher). Sixty-one percent of those who answered the question had an annual income of $75,000 or higher, with 20 patients (39%) declining to answer (see Table 1). Sixty-five percent of interviewees felt the “portal” prevented them from a face-to-face office visit, and 42% felt it prevented an urgent care or emergency room visit during the pandemic. We initially analyzed data by cohort (age, gender, caregiver) to determine how trust may be experienced differently across demographic groups; however, we were not able to detect meaningful qualitative differences. Thus, we present findings pertaining to the overall sample.

**Table 1:** Participant Characteristics

Variable	Category	Interviewee demographics (*N*=51)	2020 clinic demographics (*N*=7,867)
Mean age in years		53.2 (SD 22.5)	**-**
Mean years with clinic		6.1 (SD 6.6 )	**-**
Race	White	84% (43)	84% (6608)
	Hispanic or Latino	8% (4)	6% (527)
	Black or African American	4% (2)	4% (332)
	Asian/Pacific Islander	4% (2)	3% (237)
Highest grade or year of school completed	High school graduate, diploma or the equivalent	2% (1)	-
	Some college credit, no degree	14% (7)	-
	Associate degree	2% (1)	-
	Bachelor’s degree	41% (21)	-
	Master’s degree	37% (19)	-
	Doctorate degree	4% (2)	-
Total yearly household income*	$25,000–$34,999	2% (1)	-
	$35,000–$49,999	4% (2)	-
	$50,000–$74,999	18% (9)	-
	$75,000 or more	37% (19)	-
	Don’t know/prefer not to answer	39% (20)	-

*Thirty-nine percent of the sample (*n* = 20) did not provide income information

**Figure 1:**
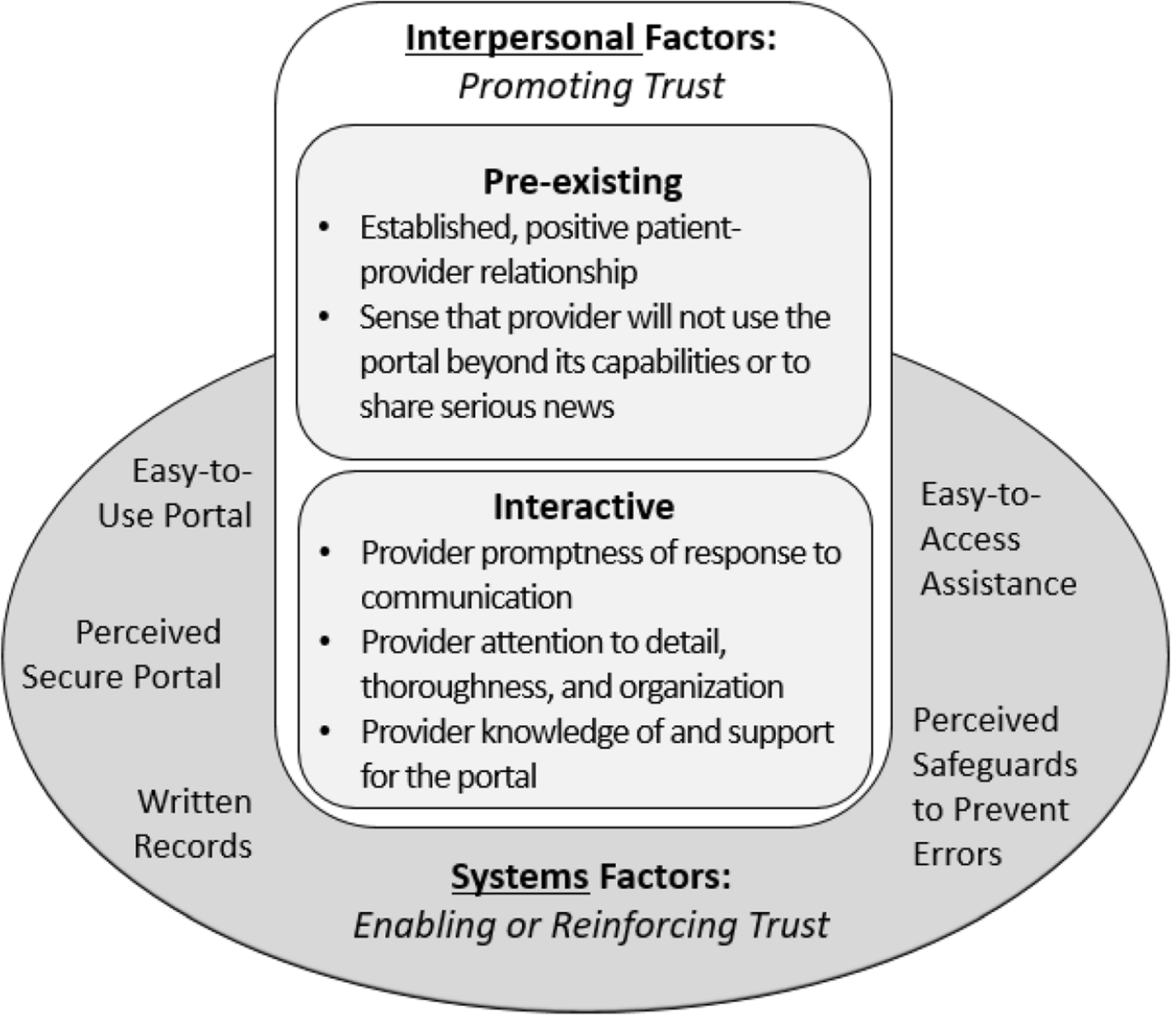
**Factors enhancing patient trust in electronic communication.**

Our principal findings center on a range of factors elucidating how patients experience trust in healthcare when it is delivered via an electronic portal. This includes both telehealth visits and less formal patient-provider communication such as email messaging and test results conveyed via the portal. Emergent themes can be classified into two general categories: interpersonal factors and systems factors (see Fig. 1). *Interpersonal* factors are those which concern patient-provider dynamics and directly promote trust. They may be *preexisting* and therefore relate to an ongoing relationship, or they may be *interactive* and pertain to patient-provider exchanges during a given encounter. In contrast to interpersonal factors, *systems* factors are those which relate to the portal itself. Each of these categories and the themes within them are described below. Illustrative quotes are provided in Table 2.

**Table 2:** Quotes Illustrating Factors Enhancing Patient Trust in Electronic Communication

Theme	Illustrative Quotes (Selected Examples)
Established, positive patient-provider relationship	“I have a lot of trust built up in them. I just transferred that trust to the [portal] app. So I have quite a bit of confidence in it.” “We’ve have had quite a long relationship... She’s very familiar with mom and always has that best interest in heart... meeting her personally over the last couple years we realized her authenticity and integrity.”
Sense that provider will not use the portal beyond its capabilities or to share serious news	“I wouldn’t do telehealth, obviously, all the time or if there was something that needed to be seen in person. But things that would be super easy to take care of online, I would feel comfortable ~ putting my trust [in the portal] as far as that goes”. “I’d imagine she’d want to also tell me in person in [the case of a serious diagnosis] … I don’t think that’s a good use through the portal. I think the portal is more to get lab results, get basic information on your health, test results coming in… I think you still need the in-person analysis for something more serious or complicated.”
Provider promptness of response to communication	“It makes me feel more confident in her because she’s very quick to reply and seems to understand what I’m trying to ask her or tell her.” “I feel my trust in my provider is increased just by knowing that they’re going to respond to me within that day or two, they’re going to be very honest in their responses.”
Provider attention to detail, thoroughness, and organization	“I think she will do what’s best because she does give me in-depth explanation. I never felt like I was short-cutted by her email. She was always thorough with me on that. So… I am pretty comfortable with her putting it through the app.” “It seems like she and her team, or whoever helps her with that, they keep her pretty well organized.”
Provider knowledge of and support for the portal	“His knowledge of the [portal] site—and he could tell me what I needed to do and where I would see the results and where I could send him messages. So he knew how to use it. We’ve used it really well together.” “I feel like she does consistently use it, which makes me feel like she trusts the system… And she believes that if that’s the way I want to communicate, that’s the way she’s going to communicate with me. She values that that’s my choice.”
Easy-to-use portal	“It feels very tidy. It feels like I don’t have to keep a bunch of files... I feel like it’s all going to be there in this place. And I can get to it anytime… the record keeping is a real plus.” “The portal has been really easy and a great way to connect. I'm always with the test results. She sent me the information back, and I can review it and ask her questions very easily.”
Perceived secure portal	“I’m very comfortable. Very confident in the process… I feel that it’s secure. I don’t think that there would be any issue with me posting something to her that’s sensitive.” “I don’t ever feel like it’s an issue… I trust it… I feel like it’s secure. It should be HIPAA-compliant.”
Written records	“I think about it more and have time when I’m writing… That’s why I like the app. I can go back and see what I wrote and make sure I didn’t miss something or make sure I said it right.” “I like when my doctor responds in email version because then I have a written explanation of what he would have originally told me in a doctor’s appointment. I find it really helpful.”
Easy-to-access assistance	“I’m sure there’s an option on the app that says “support,” “technical support”... I feel like it’d be pretty easy to just call the office if I felt the app wasn’t doing what I needed it to do.” “I could very easily [access assistance]. I’ve never had to do that, but all the options on your app and your websites are very clear and upfront so that you're not having to dig deeply.”
Perceived safeguards to prevent errors	*As patients were asked if they worried about being mistaken for other patients via the portal:* “I just have a lot of trust in my doctor and care provider team. I know that there’s a lot of identifying information that I’ve provided to the application, including pictures of myself… that I’d just assume would make it pretty easy for the doctor to distinguish between patients and not make a mistake like that.” “No, I don’t… well, I haven’t experienced that before. And it seems like lots of my identifying information is connected with my account, and so it seems like it would be pretty—maybe difficult to mix my information up with someone else’s.”

### Interpersonal Factors

Interviews elicited information on five interpersonal factors that promote trust: an established, positive patient-provider relationship; a sense that the provider will not use the portal beyond its capabilities or to share serious news; provider promptness of response to communication; provider attention to detail, thoroughness, and organization; and provider knowledge of and support for the portal, as described below.

#### Established, Positive Patient-Provider Relationship

Several interviewees expressed the importance of having an established, positive relationship with their provider and felt that face-to-face relationships “transferred” easily to the portal. Indeed, many interviewees reported ongoing interactions with their provider that had spanned several years (mean 6.1 years). Having that prior relationship appears to have eased many possible complexities of portal communication and contributed to patient trust. While our qualitative data do not indicate the *extent to which* a prior relationship is essential to promoting trust via electronic communication, a pre-existingface-to-face relationship appears helpful.

#### Sense That Provider Will Not Use the Portal Beyond Its Capabilities or to Share Serious News

Many interviewees expressed a high level of trust in care delivered through the portal but preferred that care or communication *not* be delivered electronically when a better pathway existed. In other words, patients were generally supportive of using electronic communication for certain health concerns but only “as far as that goes.” This was especially true for telehealth, which some interviewees perceived as quite limited in its clinical capabilities. Importantly, interviewees often trusted—and valued—that their provider would know which specific types of care or communication should be provided in person rather than via electronic communication.

In addition, interviewees shared their experiences with and preferences for receiving difficult diagnoses such as cancer via the portal. They overwhelmingly desired to receive serious news in person or over the phone rather than via the portal and trusted that their provider would not use the portal for such purposes. A handful of interviewees were content with the idea of receiving serious news via the portal—if it facilitated the expeditious delivery of results—but nonetheless expressed an interest in conversing with their provider shortly after receiving the results.

#### Provider Promptness of Response to Communication

More than half of interviewees valued being able to reach their provider quickly and directly and especially appreciated prompt responses to communication from their provider. Promptness not only satisfied patients in general but also led to a heightened sense “that they care.” Indeed, providers’ prompt replies were among the most salient factors contributing to a trusting relationship. Caregivers were most appreciative of the readily available “life-line” the EHR messaging and video visits provided them for loved ones during the COVID-19 pandemic.

**Figure 2:**
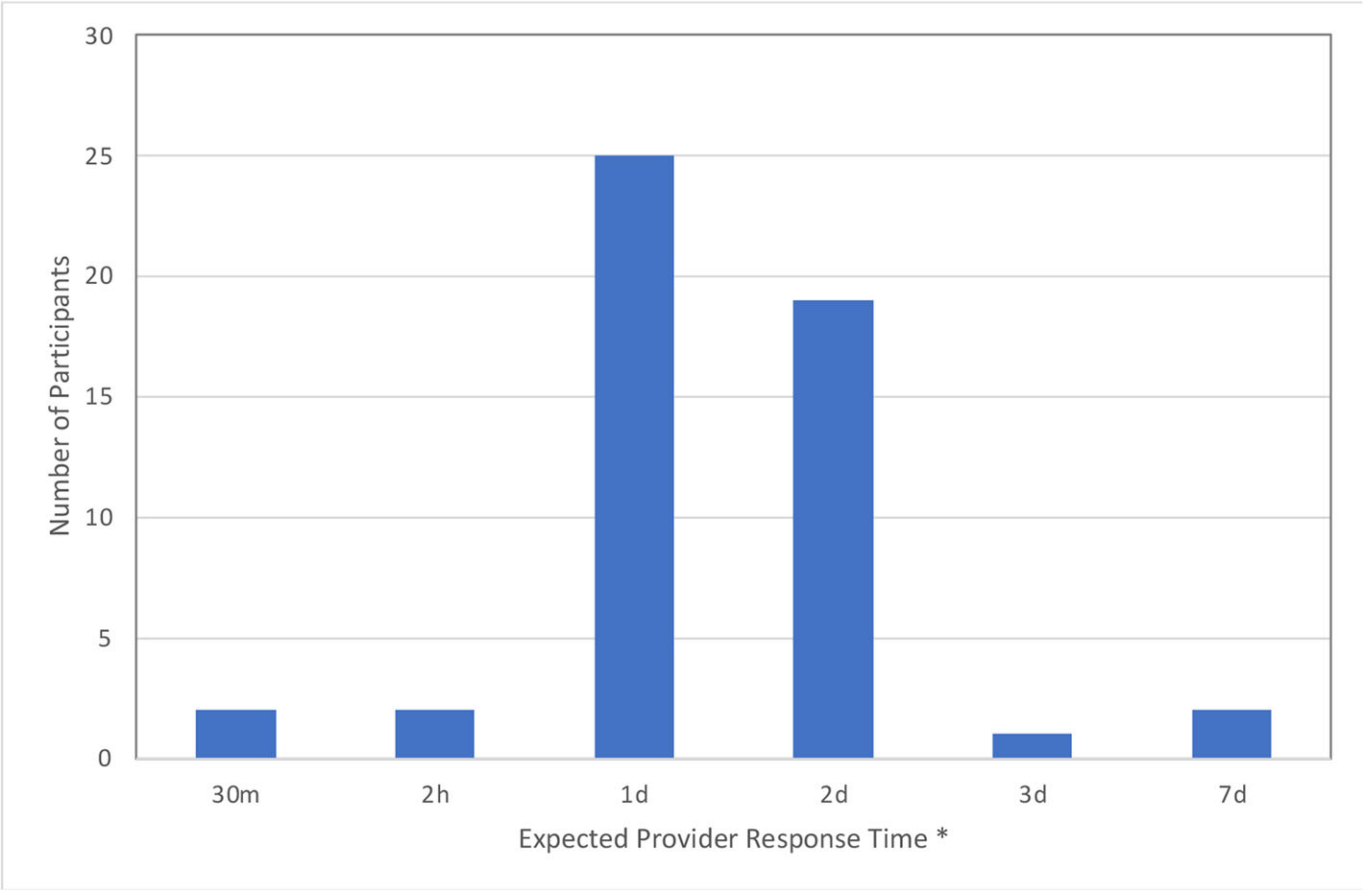
Expected provider response time to portal communications as reported by study participants (*n*= 50). *Expected response time was asked as an open-ended question and then coded.

Our data also indicate how quickly participants expect their provider to respond to communication via the portal. While a majority of patients anticipated a response within 1 day, their expectations ranged greatly from 30 min to 7 days (see Fig. 2).

#### Provider Attention to Detail, Thoroughness, and Organization

Several interviewees mentioned the importance of having a provider who is methodical in communicating and providing care via the portal. This emerged as fundamental to a trusting relationship and was described by interviewees as keen attention to detail, thoroughness, and organization. These three qualities helped patients to feel cared for, listened to, and as though they were “in good hands.” All patient messages are received directly by the physician in this clinic.

#### Provider Knowledge of and Support for the Portal

Most interviewees felt that their provider was familiar with and supportive of the portal technology, and many began using the portal at their provider’s suggestion. Meanwhile, a handful of interviewees noted that their comfort with and trust in the technology stemmed—at least in part—from their provider’s embrace of the portal. Patient confidence was connected to a perception that their provider “trusts the system.” Conversely, one patient noted reservations about the portal because of their provider’s lack of familiarity with it.

### Systems Factors

Interviewees cited several systems factors that helped to enable or reinforce trust: easy-to-use portal; perceived secure portal; written records; easy-to-access assistance; and perceived safeguards to prevent errors, as described below.

#### Easy-to-Use Portal

Overall, interviewees tended to find the portal intuitive and user friendly. However, a good handful of interviewees expressed minor difficulties such as login issues, site navigation challenges, or problems with the app freezing. Usability complications appeared to factor into decisions about how or to what extent they used the portal.

#### Perceived Secure Portal

An overwhelming majority of interviewees felt comfortable sharing personal health information via the portal and reported sharing the same information (more or less) that they would share either in person or via telephone. Interviewees valued, for example, the secure feel of the website (i.e., the login procedures) and compliance with Health Insurance Portability and Accountability Act (HIPAA) laws. A few patients preferred to discuss sensitive topics in person or via telephone rather than through the portal, but this preference appeared to be related to interpersonal dynamics rather than concerns about security.

#### Written Records

Several interviewees expressed the importance of being able to access a written record of their provider’s verbal assessment or guidance. In other words, the portal facilitated written communication with one’s provider—either in lieu of or in addition to verbal communication—and this was a valued feature. Written records helped not only patients to remember their provider’s guidance and the details of test results but also their own communication *to* their provider.

#### Easy-to-Access Assistance

High patient confidence in being able to access portal assistance was common among interviewees, and this appeared to coincide with overall comfort communicating via the portal. Despite valuing this feature, many interviewees had never accessed help.

#### Perceived Safeguards to Prevent Errors

Interviewees possessed a high degree of confidence in their provider’s ability to avert errors such as patient mix-ups via the portal. This assurance appeared to reinforce broader themes of trust in the care or communication received electronically.

## DISCUSSION

This study contributes to early research exploring trust formation and enhancement among patient portal users. Our findings complement extant research on patient portals, demonstrating these technologies can influence patient engagement and care^4^, and further define a range of interpersonal and system factors likely to facilitate or hinder optimal portal use^19^. Our results illustrate factors that promote, enable, or reinforce patient trust of electronic communication, and that pre-existing (prior to electronic encounters), interactive (during electronic encounters), and contextual (system) factors are key to a trusting patient-provider relationship. Implications are extensive.

Our results suggest potential challenges in patient trust relative to the impact of the Final Rule of the 21^st^ Century CURES Act^20^, which relates to the use of health information via electronic channels such as patient portals. Among the rule’s many provisions is a requirement that results be released to patients immediately as available, often without prior physician review. Many patients in our study noted reluctance to obtain “bad news” via the portal without explanation or advice from a provider, a finding also noted by prior research^21^.

Important policy and ethical challenges pertain to the widespread adoption of EHRs to not further widen healthcare disparities for patients less technologically literate or unable or reluctant to communicate via electronic devices^22^. While clinic demographics did not support this study to assess disparities explicitly, our data defines a range of individual and organizational factors that can inform future research on how and to what extent patients’ trust and decisions to utilize care electronically vary among broader demographic groups.

Our findings indicate a potential “uncertainty” gap, wherein patients determine the “right” way to contact their healthcare team, due to increasing choices of electronic messaging, video, telephone, or clinic visits. This was an issue for patients regarding the portal in general and telehealth specifically. A handful of patients expressed uncertainty about the extent telehealth could or should fit into their overall care.

Finally, healthcare workforce and workflow redesign efforts are beginning to evaluate staffing and space to support a “post technology” ambulatory care model with changing—and often significant—workload demands and time constraints^23^. There is an urgent need to address the burnout many physicians, especially those in primary care, are feeling with the additional time requirements of EHR in-basket tasks and patient messages^24^. Patients expect prompt and often in-depth responses to electronic communication from providers, underscoring this need to address physician workload.

This study has several strengths and limitations. First, a major strength and possible limitation is that we conducted the study amid the COVID-19 pandemic, potentially impacting responses. This unique research timing allows us to be one of the first to report on patient experiences of electronic communication at a time when face-to-face care was deemed dangerous by many patients. However, it is unclear whether our findings are transferrable to a non-pandemic environment, wherein public acceptance of (or demand for) electronic communication may be different. Second, our caregiver sampling was subject to greater potential bias than other recruitment methods since we relied on physician input for caregiver identification. Conceivably, physicians may have selectively referred only certain caregivers. Third, despite a reasonably large sample size for a qualitative study, we were not able to determine differences across participant cohort (age, gender, caregiver), therefore limiting any comparative conclusions. Finally, our participants were relatively homogenous in terms of socioeconomic status as well as race and ethnicity, and this limits the transferability of our results to diverse patient populations. Research on socioeconomic factors is limited but suggests that individuals with a college education (vs. no college degree) are more likely to access their medical record through an EHR patient portal. This underscores the need to ascertain and understand more fully how socioeconomic factors may impact portal use and trust^25^. In addition, research increasingly documents differences in trust (and mistrust) of the US healthcare system by various racial and ethnic groups^26, 27^. It is plausible that a general lack of trust, perhaps in conjunction with other access barriers, may translate to disparities in EHR portal use. Current evidence is mixed and inconclusive^25, 28, 29^, and therefore it is crucial that future research assesses how electronic portals are perceived and experienced by patients from diverse racial and ethnic groups.

Overall, our study provides key insights into themes of patient trust and indicates that the portal is a highly valued communication tool that can enhance trust and engagement when used optimally for some patients. Important policy work, expanded demographic research, and patient and healthcare professional education remain to be done to support optimal use to understand and maximize this technology’s potential.

## Supplementary Information

ESM 1 (PDF 39 kb)
