# Supplementary material for: Factors Enhancing Trust in Electronic Communication Among Patients from an Internal Medicine Clinic: Qualitative Results of the RECEPT Study
Source: J Gen Intern Med. 2022 Jan 19;37(12):3121–7. doi: 10.1007/s11606-021-07345-9 (PMC8768442; doi:10.1007/s11606-021-07345-9)
Supplement: Supplementary file 1 — (PDF 39 kb) [file 11606_2021_7345_MOESM1_ESM.pdf]

Supplementary Appendix: Codes and Categories Used In Data Analysis

**Initial Codes**	**Categories**
ED or urgent care prevention: [Yes, No, Mixed/Other]	Appointment Prevention
In-person appointment prevention: [Yes, No, Mixed/Other]	Appointment Prevention
Too complex for email	Appointment Prevention
Impact of COVID-19 on patient	COVID-19
Using telehealth only during COVID-19	COVID-19
Frequency of use	App/Portal Use Details
Impetus: [Provider, Self, Unsure/Unclear]	App/Portal Use Details
Modality: [App, Website, Both]	App/Portal Use Details
Provider support for portal: [Yes, No, Mixed/Other]	App/Portal Use Details
Time frame of use: [Less than 1 Year, More than 1 Year]	App/Portal Use Details
Help: [Knows How to Access, Not Sure How to Access, Mixed/Other]	Features Used or Not Used
Features used: [Attachment, Away, E-Consult, Financial, Help, Other, Reminder, Results, Scheduling, Telehealth]	Features Used or Not Used
Features NOT used: [Same options as above]	Features Used or Not Used
Features most valued: [Same options as above]	Satisfaction and Ease of Use
Satisfied with app or portal: [Yes, No, Mixed/Other]	Satisfaction and Ease of Use
Challenge: Completing forms or consent	Satisfaction and Ease of Use
Challenge: Difficulty obtaining prescriptions	Satisfaction and Ease of Use
Challenge: Explanation of billing or financial information	Satisfaction and Ease of Use
Challenge: Finding or accessing app or portal	Satisfaction and Ease of Use
Challenge: Medical jargon or abbreviations	Satisfaction and Ease of Use
Challenge: Self-monitoring	Satisfaction and Ease of Use
Challenge: Technological	Satisfaction and Ease of Use
Challenge: Lack of time	Satisfaction and Ease of Use
Completeness and quality of information to patient: [Good, Not good, Mixed/Other]	Satisfaction and Ease of Use
Easy to use: [Difficult, Easy, Mixed/Other]	Satisfaction and Ease of Use
Expectation for response - General message: [Varies, 1-2 Days, 3-5 Days, 6-7 Days 7+ Days]	Satisfaction and Ease of Use
Expectations for response - Test results: [Varies, 1-2 Days, 3-5 Days, 6-7 Days 7+ Days]	Satisfaction and Ease of Use
Features desired	Satisfaction and Ease of Use
Instructions: [Sufficient, Not sufficient, Mixed/Other]	Satisfaction and Ease of Use
Recommendation	Satisfaction and Ease of Use
Satisfaction with responsiveness: [Satisfied, Not Satisfied, Mixed/Other]	Satisfaction and Ease of Use
Satisfaction with telehealth: [Satisfied, Not Satisfied, Mixed/Other]	Satisfaction and Ease of Use
Values written record	Satisfaction and Ease of Use
Challenge: Internet privacy	Trust
Challenge: Worry that health issue may be overlooked or misdiagnosed	Trust
Comfort sharing private information: [Comfortable, Not Comfortable, Mixed/Other]	Trust
Preference regarding difficult diagnosis via portal: [Receive, Not Receive, Mixed/Other]	Trust
Perceived provider honesty via portal: [High, Neutral to Negative, Mixed/Other]	Trust
Concerned about mix-up with other patients: [Worried, Not Worried, Mixed/Other]	Trust
Trust via app or portal: [High, Neutral to Negative, Mixed/Other]	Trust
**Focused Codes**	**Categories**
Trusting because of provider promptness of response to communication	Interpersonal/Interactive Factors
Trusting because of provider attention to detail, thoroughness, and organization	Interpersonal/Interactive Factors
Trusting because of provider knowledge of and support for portal technology	Interpersonal/Interactive Factors
Trusting because of an established, positive patient-provider relationship	Interpersonal/Preexisting Factors
Trusting because of a sense that provider will not use the portal for certain purposes	Interpersonal/Preexisting Factors
Trusting because of perceived safeguards to prevent errors	Systems Factors
Trusting because of written records	Systems Factors

Trusting because of perceived secure portal	Systems Factors
Trusting because of easy-to-use portal	Systems Factors
Trusting because of easy-to-access assistance	Systems Factors
